# Climate scientists set the bar of proof too high

**DOI:** 10.1007/s10584-021-03061-9

**Published:** 2021-04-19

**Authors:** Elisabeth A. Lloyd, Naomi Oreskes, Sonia I. Seneviratne, Edward J. Larson

**Affiliations:** 1 History and Philosophy of Science and Medicine Department, Indiana University, Bloomington, IN USA; 2 History of Science Department, Harvard University, Cambridge, MA USA; 3 Institute for Atmospheric and Climate Science, ETH Zurich, Zurich, Switzerland; 4 Humanities Division and Caruso School of Law, Pepperdine University, Malibu, CA USA

**Keywords:** Extreme event attribution, Standards of proof, Policy and evidence, Legal contexts of climate attribution, Science communication

## Abstract

Standards of proof for attributing real world events/damage to global warming should be the same as in clinical or environmental lawsuits, argue Lloyd et al. The central question that we raise is effective communication. How can climate scientists best and effectively communicate their findings to crucial non-expert audiences, including public policy makers and civil society? To address this question, we look at the mismatch between what courts require and what climate scientists are setting as a bar of proof. Our first point is that scientists typically demand too much of themselves in terms of evidence, in comparison with the level of evidence required in a legal, regulatory, or public policy context. Our second point is to recommend that the Intergovernmental Panel on Climate Change recommend more prominently the use of the category “more likely than not” as a level of proof in their reports, as this corresponds to the standard of proof most frequently required in civil court rooms. This has also implications for public policy and the public communication of climate evidence.

**Supplementary Information:**

The online version contains supplementary material available at 10.1007/s10584-021-03061-9.

## Introduction

Consider our situation with the coronavirus. We often have to make a variety of policy, practical, and legal decisions based on incomplete information, which also depend on judgements about whether the evidence is good enough. What level of evidence do we need, in the case of the coronavirus, to order a stay-at-home command for an entire city or state? What is the level of evidence required to actively prepare for catastrophic needs for intensive care units in hospitals? If there is an immediate and/or grave threat, as we have seen, it may be better to act on a lower level of evidence than we might otherwise expect. Or consider the epic tobacco litigation over the past quarter century in the USA. Court after court found that American tobacco companies knowingly or negligently produced or marketed cigarettes in a manner that caused foreseeable harm to millions of Americans resulting in billions of dollars of medical costs and individual damages. Proving each element of the case by a preponderance of evidence was seen as enough in these cases. In short, was the injury more likely than not caused by the tobacco company’s knowing or negligent acts?

Given the impending threats of climate change, we are also faced with incomplete or imperfect information, but we do have enough to make judgments in a variety of policy, pragmatic, and legal contexts. In the climate case, we think scientists have often been setting the evidential bar too high, given the actual level of threat.

Climate scientists generally look for a probability of 90–100% before they call a scientific claim—the attribution of a global heat record to climate change, say—“very likely.” This is what is done in reports of the UN’s Intergovernmental Panel on Climate Change (IPCC) (e.g., IPCC 2013, 2018; Seneviratne et al. 2012; Hoegh-Guldberg et al. 2018), based on their 2010 guidance document on the treatment of uncertainties (e.g., Mastrandrea et al. 2010, 2011). The legal profession often requires demonstration at a much lower level. In the USA, for example, the standard for a civil case in medical malpractice or patent infringement, to cite two quasi-scientific instances, is “more likely than not,” generally interpreted as a probability of more than 50%. We hence argue that the “more likely than not” (greater than 50%) category that was sidelined in IPCC uncertainty language by the 2010 guidance document (Table 1) would be highly appropriate in many legal contexts, where it is essentially identical to the standard used in US courts for medical malpractice, patent infringement, and civil tort claims.

**Table 1:** Likelihood scale based on the uncertainty guidance from IPCC (Mastrandrea et al. 2010). Permission granted for public use by IPCC

Likelihood scale	
Term^*^	Likelihood of the outcome
Virtually certain	99–100% probability
Very likely	90–100% probability
Likely	66–100% probability
About as likely as not	33 to 66% probability
Unlikely	0–33% probability
Very unlikely	0–10% probability
Exceptionally unlikely	0–1% probability

Additional terms that were used in limited circumstances in the AR4 (*extremely likely* 95–100% probability, *more likely than not* > 50–100% probability, and extremely *unlikely* 0–5% probability) may also be used in the AR5 when appropriate

In other words, climate scientists have set themselves a higher level of proof in order to make a scientific claim than law courts ask for in civil litigation in the USA, the UK, and virtually all common law countries. In US tort law, as just one example, plaintiffs typically must show that their individual injuries were “more likely than not” caused by the behavior in question. This requirement has been met through showing that the behavior in question increased the risk of the harm occurring by a factor of two, with this two-fold factor addressing the issue of causation rather than proof (Burger et al. 2020, p. 206). The UK applies an effectively equivalent “more probable than not” standard of proof for actions holding individuals and corporations civilly liable for their tortuous actions (in Re B (Children0 [2008] UKHL 35 “Neither the seriousness of the offence nor the seriousness of the consequences should make any difference to the standard of proof to be applied”). Civil cases may be lost or won simply because of a gap between scientific and legal standards.

A possible objection is that the standard of “more likely than not” or “more probable than not” increases the risk of type I errors—attributing causation where it is not warranted—as compared with adopting a higher bar. That is true. But as shown elsewhere (Winsberg et al. 2020, Lloyd and Oreskes 2018; Mann et al. 2017), all decisions about standards of proof involve judgments as to the relative risks of type I and type II errors. Our point here is not to assert that one type of error is worse than the other, but rather to point out that the scientific standard, “more likely than not,” most closely approaches the legal standard that will be applied in many, if not most, climate change lawsuits.^1^ Therefore, with respect to issues of climate detection and attribution that may end up being salient in such lawsuits, scientists should have available to them—and, we argue, in many cases appropriately invoke—that standard.

Our argument is also pertinent to the problem of communication. If scientists want to better communicate their knowledge in a way that the lawyers, judges, and public policy workers will understand as intended, these are our recommendations: In our view, the too narrow focus of climate science on extremely stringent levels of proofs is damaging in a legal context and can lead to confusion when communicating scientific findings more generally. We argue that standards for scientific proof for climate science should be considered that are appropriate for use in a specific context, a point that has been emphasized in philosophy of science by some (Lloyd 2015; Knüsel et al. 2020; Parker and Lusk 2019; Winsberg 2018), much as scientific and clinical standards for causality are used in tort law contexts. For example, to prevail in a tort action against the producer of a toxic chemical, such as asbestos, the injured party only needs to show that it is more likely than not that the producer’s negligence caused the injury. Furthermore, we propose that a category of “more likely than not (> 50%)” (or what in most common law countries is called “the balance of probabilities standard”) is most appropriate for use in a wide range of contexts and call upon the Intergovernmental Panel on Climate Change to adapt their current recommendations (see Table 1), and apply it more widely in their upcoming reports.

## Never proven: Climate evidence in IPCC reports

What does it take to prove a scientific claim? Scientists and philosophers have grappled with this issue for centuries, but the difficulty of resolving it has been exacerbated in recent years as interested parties exploit scientific uncertainty as justification for policy inaction (Oreskes and Conway 2010). In response, in the climate change arena, scientists have labored both to reduce the uncertainties and to be diligent about articulating—and where possible quantifying—them. Arguably, this is one reason that IPCC reports have become longer and longer over time, as scientists strive to make sure that all possible complaints and objections are fully addressed. It is also a reason why scientists and others have attempted to quantify the degree and components of consensus on key questions of anthropogenic climate change (Oreskes 2004; Doran and Zimmerman 2009; Maibach et al. 2014; Cook et al. 2013, 2016).

IPCC reports, which are the results of writing and reviews by thousands of scientists, also include consensus statements in their “Summary for policymakers” which are approved by government delegations, under validation by the involved scientists. The result of these reports is that the climate science community has made clear that human-caused emissions have changed the climate profoundly, which is known beyond reasonable doubt.

Articulating the level of certainty of a knowledge claim as significant, is useful in part because policy decisions involve both risk assessment of damages based on the science, and the judged credibility of the underlying science. Different scientific claims may have different risks to society or individuals attached to them. The risk to society of manufacturing a faulty belt buckle is vanishingly small, while the risk of damages from many extreme climate events is large; weighing those risks of damages helps people decide whether the level of confidence in a claim is enough to justify taking that risk. But we must also weigh how conclusive the claim of potential large damage is. Indeed, the greater the claim of expected damage, the more some people will be inclined to find the claim “not” credible. Likewise, the more costly the required policy intervention, the more some people will resist accepting its necessity. Therefore, the certainty of the science underlying the claim is a relevant factor in decision-making.

As long as the evidence for a hypothesis is not 100% conclusive, we cannot accept or reject the hypothesis without balancing the risk of falsely rejecting the hypothesis against the risk of falsely accepting it. As Winsberg et al. (2020) note, “In the case of climate science, those principal risks, as everyone knows, are the risks of being inadequately prepared for, or inadequately mitigating, the increased damages and lives lost produced by severe weather events, on the one hand, and the risk of over-preparing or over-mitigating on the other” (p. 10).

Proof in the climate change context is particularly urgent for two reasons: one is that there are legal cases that hinge in part on whether anthropogenic climate change is proven, and two because we are running out of time (see the IPCC’s recent 1.5 degree report (2018)). If the scientists are right, we have only a decade to act decisively to dramatically bend the emissions curve to put us on track to achieve the Paris Agreement’s aims.

One issue common to all or most legal cases is the establishment of a causal connection between global climate change and specific harms to individuals or groups in particular regions, claims that are established through extreme event attribution studies (e.g., cases won, lost, and in progress include: Greenpeace Southeast Asia and Philippine Rural Reconstruction Movement 2015; *Urgenda Foundation v. The Netherlands* 2018; Juliana et al. v. U.S.A. 2018; *County of San Mateo v Chevron Corp* et al., 2018; Union of Senior Swiss Women for climate protection vs. Swiss Federal Parliament 2018). This is where the two-fold causation standard mentioned above comes into play to assure that causality is firmly established. With this protection in place, the standard common law burden of proof is appropriate.

To those working outside of the field, it may seem that climate scientists have set for themselves a standard in which *nothing* is ever proven. As part of the framework of the Intergovernmental Panel on Climate Change (IPCC), the highest level of proof with 99–100% probability is characterized as “virtually certain” (Table 1). Even at the 100% level, scientists avoid the word proven, seemingly leaving room for doubt and, for climate change issues, the 99% certainty standard for individual actions is unrealistic. This can be problematic when scientific findings are discussed in court or with policy makers. But, as with the coronavirus pandemic, all kinds of decisions need to be made with imperfect information, with uncertainty and under times of urgency, including the urgency of climate change damages.

For example, during legal battles over compensation for damages as a result of human-caused climate change—such as a case involving demonstrable injury flowing from identifiable acts by a particular defendant—witnesses can insist that key tenets of climate science are not proven, even when most scientists consider that they are, in fact, proven to all intents and purposes. In the climate context, such cases could involve the emission of greenhouse gases above the applicable legal limits or unauthorized forest destruction. In many countries, the risk of alternative civil liability could prove more effective that the possibility of criminal liability, with the two-fold causality standard in place to protect against abusive lawsuits.

The debates over lead poisoning, tobacco, or water are tort cases involving damages, and concern whether the science is sufficient to show that damage is really there. In a legal setting, the standard of evidence for a civil case is clear: the preponderance of evidence, or greater than 50%. But when scientists look at an issue, the standard they ask of themselves is not justified in this context, because they ordinarily demand a 95% standard in order to make a scientific claim (a focus also recently criticized by statisticians, e.g., Amrhein et al. 2019). It is this difference between what courts accept and what scientists are demanding of themselves and other scientists that concerns us in this commentary.

We suggest that scientists should match standards of demonstration to the problem at hand and the context in which their findings are being used. Moreover, we believe that, in the context of scientific assessment for public policy and civil litigation, a standard that corresponds to or is even higher than the level of proof required for a criminal conviction is logically inappropriate and potentially harmful to the victims of climate change damage, who may end up denied compensation for tangible harms.

## Balance of evidence: What is required in legal context

Advances in the science of detection and attribution, especially analysis of extreme events such as heat waves, have allowed such analyses to be used as evidence in legal cases involving climate change (see in-depth treatment in Burger et al. 2020). Climate models aid in the attribution of extreme events both through the probabilistic and storyline or mechanistic methods (Lloyd and Oreskes 2018; Shepherd 2019); in both cases, extreme events such as heat waves or heavy precipitation events can generally be attributed to climate change with a high degree of confidence. Such attributions are then useful to establish standing to allow a person or persons to sue in court, and for tort cases in which emitters are held liable for damages from climate change. The typical attribution studies presented in court have confidence levels of greater than 90%, many having a confidence level of > 95%, far exceeding the legal standard required by the courts in these cases (Burger et al. 2020, p. 170).

An important context in which IPCC science provides information is in courtrooms where damage claims from anthropogenic climate change are pending. For example, in Clean Air Act litigation, there is a case, *Coalition for Responsible Regulation v. EPA*, where an industry group argued that a previous Court ruling, in *Massachusetts v. EPA*, had relied too much on evidence from the IPCC and other scientific organizations. This was not a winning argument, and the court deciding the case found that the climate science evidence showed sufficient grounding of the relevant causal claims from the IPCC. Similar legal standards held in the *Juliana* et al. *v*. *USA* case, where the plaintiffs needed to show a causal connection between their loss of human and constitutional rights, standing, and merits claims, and the US government-approved emissions. Burger et al. (2020, p. 179) note that much can be learned from the preparation done regarding the attribution claims of the *Juliana* case, as it is a model for future applications of detection and attribution science. In each instance, the court will decide the case following the civil legal standard of “more likely than not.” So can researchers support a link between global climate change and, for example, fires or floods that may have caused damage or harm, as K. Trenberth did for the *Juliana* case? And what standard of proof should be applied in court for the scientific work?

Recent advances have, in fact, enabled scientists to build a chain of causality between trends in extreme events and human-induced global warming, even on regional scale (Zwiers et al. 2011, Seneviratne et al. 2012, Bindoff et al. 2013, IPCC 2018, Hoegh-Guldberg et al. 2018; Table 1 in Supplementary Material). In addition, the contribution of anthropogenic global warming to the probability of a heatwave, heavy precipitation event, or drought say, can be now robustly assessed, contrary to the denials presented in *Juliana v. USA* by defense witness Weyant (e.g., Stott et al. 2004, NAS 2016).

But what degree of specificity is necessary in a court case? This is where the question of standards of proof comes into play.

A recent attribution study (Knutson et al. 2019) about tropical cyclone intensity found that the > 50% or “more likely than not” standard common in civil court cases altered scientific conclusions substantially from using the > 95% standard. The study looked at whether changes in cyclone activity could be detected, and whether any detected changes could be attributed to anthropogenic global warming. The researchers examined the effects of either avoiding false positive (type 1) errors (overstating influence or detection) or false negative (type II) errors (missing or understating influence or detection).

In addition, for detecting climate change, they examined two standards of scientific evidence, the usual 90% and the over 50% level, which is compatible with the legal context of civil cases in the USA, the UK, and commonwealth countries.

Using the conventional scientific approach of avoiding false positive errors and using the > 95% standard, they found low confidence in the detection and attribution of any anthropogenic influence on the intensity of tropical cyclones in any basin. In contrast, when reducing false negative errors and using the balance of evidence > 50% standard, with each co-author making their own judgement, ten of 11 authors concluded that there is *a detectable increase in the global average intensity of the strongest hurricane strength cyclones* in the tropics since the early 1980s. On the issue of the attribution, eight of 11 authors concluded that *anthropogenic forcing has contributed to the detected increase* in global average intensity of these most powerful of wind storms. The reduction of false positive error might be conducted purely for the sake of improving our understanding of the world, but as Knutson et al. write, “for future planning and risk assessment, one may want to reduce [false negative] errors in particular. For example, planners for infrastructure development in coastal regions may want to consider emerging detection/attribution findings—even if not at the 0.05 significance [95%] level—in their planning and decision-making” (Knutson et al. 2019). The point of such an analysis is not to assert that one approach is better than the other in any absolute sense. Rather, it suggests that the appropriate approach depends on the problem at hand and the context in which claims are being evaluated (Lloyd and Oreskes 2018; Mann et al. 2017).

## Conclusion

We suggest that the IPCC should apply routinely the category of “more likely than not” in all of its reporting contexts, recognizing its legal and thus policy relevance. The category was minimized after the Fourth Assessment Report, appearing only as a footnoted possibility in the IPCC uncertainty guidance document (Table 1); we argue that it should be recommended again as an established and routine option. This can be done by the IPCC instituting a new guidance, in which “more likely than not” is highlighted as a relevant assessment level for legal contexts to be used throughout the assessment reports wherever authors find it appropriate.

We note as a possible source of confusion that the IPCC also includes confidence levels in addition to likelihood levels (the latter being generally only applied in cases of high confidence, Mastrandrea et al. 2011), and that “medium confidence” statements can be considered as broadly equivalent to a more likely than not category (with the balance of evidence pointing to a given conclusion). Nonetheless, the explicit use of the “more likely than not” category would better parallel the nomenclature used in courts.

An additional benefit to our proposal is that, unlike many scientific and technical terms, the phrase “more likely than not” has a common sense, every day meaning that is consistent with its scientific meaning. This suggests that its use would increase the odds that the audience for IPCC information understands climate evidence as the IPCC intends it to. Indeed, our argument also applies beyond courtrooms and more generally to the public discourse on climate change.

As an area for future study, we note that some research has indicated that the language used by climate scientists to characterize their findings is often misinterpreted and viewed as conveying a lesser degree of certainty than scientists intend. In one survey, participants with no science background consistently interpreted the IPCC’s “very likely” standard as indicating a 65–75% probability, rather than the 90–100% that the IPCC intends (see Table 1; Budescu et al. 2012). In this paper, we have focused on the importance of standards of proof in relation to legal matters, but there may be a larger social benefit to scientists looking more closely at the language they use to communicate their confidence in their findings.

Scientists may object that we are asking them to “lower their standards,” and thereby, as they would see it, to weaken their science. This is not our argument. We agree that climate scientists should continue to strive for the highest possible level of scientific understanding of the climate system. However, we argue that, in the context of scientific assessments of direct relevance for public policy and civil litigation, focusing on a standard that corresponds to or is even higher than the level of 95–99% proof required for a *criminal* conviction may be inappropriate and potentially harmful. Ultimately, providing a range of possible conclusions from existing data for different levels of proofs (e.g., both at “more likely than not” and higher) would likely be the best way in which climate scientists can inform society on climate evidence, both inside and outside the courts.

## Supplementary information

ESM 1 (DOCX 17 kb)

## Data Availability

The data behind the chart in the paper is available from the IPCC, open access.

## Footnotes

1 It is often repeated in the preparation of IPCC report that we should be ‘policy relevant’ but not ‘policy prescriptive’. Using a standard of proof that is better usable in courts is policy-relevant, since policies can also be changed in courts as a result of creating precedents.
