# Supplementary material for: Climate scientists set the bar of proof too high
Source: Clim Change. 2021 Apr 19;165(3-4):55. doi: 10.1007/s10584-021-03061-9 (PMC8054254; doi:10.1007/s10584-021-03061-9)
Supplement: Supplementary file 1 — (DOCX 17 kb) [file 10584_2021_3061_MOESM1_ESM.docx]

Supplemental Material:

Table 1: Assessments of changes in extremes for observed changes and their attribution, and for projected changes at 1.5°C and 2°C of global warming, with associated likelihood or confidence levels and corresponding probabilities (based on IPCC 2018; Hoegh-Guldberg, et al., in press)

|  | Observed changes | Attribution | Projected changes at 1.5°C of global warming (unavoidable) compared to pre-industrial conditions | Projected changes at 2°C of global warming (still physically avoidable) compared to 1.5°C |
| --- | --- | --- | --- | --- |
| Hot temperature extremes | *Very likely* (≥90%) increase of hot days and nights (on global scale and on continental scale in North America, Europe and Australia) | *Very likely* (≥90%) contribution of human-induced emissions to observed changes on global scale | *Very likely* (≥90%) increase of hot days and nights  *High confidence* (>50%)* that increase would be highest over land including many inhabited regions with increases of up to 3°C in mid-latitude warm season | *High confidence* (>50%)* increase in hot days and nights compared to 1.5°C of global warming  *High confidence* (>50%)* in global-scale increase in length of warm spells and decrease in length of cold spells  *High confidence* (>50%)* that strongest increase in frequency would be for the rarest and most extreme events  *High confidence* (>50%)* in particularly large increases in hot extremes in inhabited regions |
| Heavy precipitation events | *Likely* (≥66%) more areas with increases than decreases in the frequency,  intensity and/or amount of  heavy precipitation | *Medium confidence* (>50%)* that human influence contributed  to the global-scale  tendency towards increases in  the frequency, intensity and/or amount of heavy precipitation  events | *High confidence* (>50%)* in increases in frequency, intensity and/or amount heavy precipitation when  averaged over global land, with positive trends in several regions | *Medium confidence* (>50%)* in higher frequency, intensity  and/or amount of heavy  precipitation when averaged over global land, with positive trends in several regions.  *Medium confidence* (>50%)* that several regions are projected to experience increases in heavy precipitation at 2°C versus 1.5°C, in particular in high-latitude and mountainous regions, as well as in eastern Asia and eastern North America |

**Medium confidence* assessed here as >50% given the overall balance of evidence (see text). *High confidence* would be expected to be higher (e.g. >70%) but is not linked in IPCC guidance to a specific quantitative level (Mastrandrea et al. 2011).
